# Application of Next-Generation Sequencing to Enterobacter Hormaechei Subspecies Analysis during a Neonatal Intensive Care Unit Outbreak

**DOI:** 10.3390/children10101696

**Published:** 2023-10-16

**Authors:** Patrick Morhart, Roman G. Gerlach, Caroline Kunz, Jürgen Held, Giuseppe Valenza, Joachim Wölfle, Heiko Reutter, Gregor J. Hanslik, Fabian B. Fahlbusch

**Affiliations:** 1 Division of Neonatology and Paediatric Intensive Care Medicine, Department of Paediatrics and Adolescent Medicine, Friedrich-Alexander-University of Erlangen-Nürnberg, 91054 Erlangen, Germany; patrick.morhart@uk-erlangen.de (P.M.); heiko.reutter@uk-erlangen.de (H.R.); gregor.hanslik@uk-erlangen.de (G.J.H.); 2 Institute of Microbiology—Clinical Microbiology, Immunology and Hygiene, Friedrich-Alexander-University of Erlangen-Nürnberg, 91054 Erlangen, Germanycaroline.kunz@gmx.net (C.K.); juergen.held@uk-erlangen.de (J.H.); giuseppe.valenza@uk-erlangen.de (G.V.); 3 Department of Paediatrics and Adolescent Medicine, Friedrich-Alexander-University of Erlangen-Nürnberg, 91054 Erlangen, Germany; joachim.woelfle@uk-erlangen.de; 4 Neonatology and Pediatric Intensive Care, Faculty of Medicine, University of Augsburg, 86156 Augsburg, Germany

**Keywords:** *Enterobacter*, MRGN, ECC, NICU, NGS, WGS

## Abstract

Introduction: The *Enterobacter cloacae* complex (ECC) species are potential neonatal pathogens, and ECC strains are among the most commonly encountered *Enterobacter* spp. associated with nosocomial bloodstream infections. Outbreaks caused by ECC can lead to significant morbidity and mortality in susceptible neonates. At the molecular level, ECC exhibits genomic heterogeneity, with six closely related species and subspecies. Genetic variability poses a challenge in accurately identifying outbreaks by determining the clonality of ECC isolates. This difficulty is further compounded by the limitations of the commonly used molecular typing methods, such as pulsed field gel electrophoresis, which do not provide reliable accuracy in distinguishing between ECC strains and can lead to incorrect conclusions. Next-generation sequencing (NGS) offers superior resolution in determining strain relatedness. Therefore, we investigated the clinical pertinence of incorporating NGS into existing bundle measures to enhance patient management during an outbreak of ECC in a level-3 neonatal intensive care unit (NICU) in Germany. Methods: As the standard of care, all neonates on the NICU received weekly microbiological swabs (nasopharyngeal and rectal) and analysis of endotracheal secretion, where feasible. During the 2.5-month outbreak, colonisation with ECC was detected in n = 10 neonates. The phylogenetic relationship and potential antimicrobial resistance genes as well as mobile genetic elements were identified via bacterial whole-genome sequencing (WGS) using Illumina MiSeq followed by in silico data analysis. Results: Although all ECC isolates exhibited almost identical antimicrobial susceptibility patterns, the WGS data revealed the involvement of four different ECC clones. The isolates could be characterised as *Enterobacter hormaechei* subspecies *steigerwaltii* (n = 6, clonal), subsp. *hoffmannii* (n = 3, two clones) and subsp. *oharae* (n = 1). Despite the collection of environmental samples, no source of this diffuse outbreak could be identified. A new standardised operating procedure was implemented to enhance the management of neonates colonised with MRGN. This collaborative approach involved both parents and medical professionals and successfully prevented further transmission of ECC. Conclusions: Initially, it was believed that the NICU outbreak was caused by a single ECC clone due to the similarity in antibiotic resistance. However, our findings show that antibiotic susceptibility patterns can be misleading in investigating outbreaks of multi-drug-resistant ECC. In contrast, bacterial WGS accurately identified ECC at the clonal level, which significantly helped to delineate the nature of the observed outbreak.

## 1. Introduction

Newborns, particularly preterm neonates, who are admitted to a neonatal intensive care unit (NICU) are highly susceptible to temporary or permanent colonisation of their skin and/or mucous membranes with multi-drug-resistant Gram-negative bacteria (MRGN). This is primarily due to the frequent administration of antibiotics and the extended duration of hospitalisation [1]. Importantly, premature infants face a higher likelihood of developing MRGN infections because of their immature immune state, especially when confronted with intestinal complications (e.g., necrotising enterocolitis) or healthcare-associated infections (HAIs, e.g., central venous catheters, parenteral nutrition, invasive ventilation and drains). Infectious material includes various substances such as respiratory secretions (including aerogenic transmission), faeces, urine and wound secretions, among others [2]. Thus, it is important to avoid the contamination of surfaces—especially of wet areas within the immediate vicinity of the patient’s environment (e.g., incubators, washbasins, syphons, taps, water baths, storage tanks of humidifiers, etc.). Surface contamination enables transmission via contaminated hands (parents/medical and nursing staff), material or food. 

In Germany, MRGN are classified according to the guidelines from the Commission for Hospital Hygiene and Infection Prevention (KRINKO) of the Robert Koch Institute (RKI, Berlin, Germany): In adults, multi-drug resistance (MDR) of Gram-negative bacilli is defined as phenotypic resistance to ≥3 of 4 antibiotic classes ([1] piperacillin, [2] third generation cephalosporins, [3] flurochinolones and [4] carbapenems) or the detection of carbapenemases. However, in neonates, a separate class, so-called 2MRGN-NeoPaed, is defined, which exhibits phenotypic resistance to two out of the four antibiotic classes [3], as the use of fluoroquinolones for empirical therapy is not suitable in neonatology and paediatrics.

In addition to aiding decision-making in antibiotic stewardship (ABS), the early detection of MRGN status plays a crucial role in implementing patient isolation and cohorting strategies in NICUs, hence the German KRINKO-published recommendations on patient-related colonisation screening (CoS) for MRGN bacilli in NICU neonates, as of 2007 [4]. These involve obligatory nasopharyngeal (or throat) and rectal swabs to facilitate a calculated antibiotic therapy at an early stage in very low-birth-weight infants of all NICUs in Germany.

Most neonates are asymptomatic and not infected upon the first MRGN detection (colonised state). Noteworthy, the infection rate of previously colonised neonates depends not only on the host-specific risk factors associated with the premature state [5,6,7] but also on the bacterial species [8]. Typical Gram-negative infectious agents associated with late-onset sepsis in neonates are *Enterobacter cloacae* complex (ECC), *Klebsiella pneumoniae* and *Serratia marcescens* [5,8,9,10,11,12]. It is of note that the role of MRGN colonisation as a potential driver of the infection of preterm neonates at later stages is currently being controversially discussed [13,14,15].

Due to the close relationships within the ECC, antibiotic susceptibility patterns (ASTs) and routine matrix-assisted laser desorption–ionisation time of flight mass spectrometry (MALDI–TOF MS) can be misleading in indicating phylogenetic relatedness within the ECC in outbreak scenarios [16], as shown by previous studies [17,18,19,20,21,22]. Thus, high-resolution strain typing methods such as pulsed field gel electrophoresis (PFGE) or whole-genome sequencing (WGS) are required [19,23,24,25]. WGS offers superior resolution compared to the previous gold standard method of PFGE, allowing for the identification of outbreaks at subclonal levels. Furthermore, the use of next-generation sequencing (NGS) data and tools for phylogenetic analyses such as core genome multilocus sequence typing (cgMLST) or the detection of whole-genome single-nucleotide polymorphisms (wgSNPs) offers the opportunity to improve outbreak investigations by providing information on the microbial community composition of the hospital environment [26,27,28]. In our study, we describe the usage of bacterial WGS in combination with wgSNP analysis as a valuable tool for the management of an ECC outbreak on a German tertiary NICU.

## 2. Materials and Methods

### 2.1. Study Population

The Department of Paediatrics and Adolescent Medicine of the Friedrich-Alexander-University Erlangen-Nürnberg, Germany, includes a 14-bed level-3 neonatal intensive care unit (NICU) with an associated standard neonatal care unit of 16 beds. A level-3 NICU is a specialised healthcare facility that provides the highest level of specialised care for critically ill newborns, including those with complex medical conditions and those requiring advanced interventions. In 2020, the NICU had 799 admissions from both in-born and referred patients. Along with its associated children’s heart centre and pediatric intensive care unit, it functions as a reference centre for other hospitals in the Franconia province of Bavaria. Bacterial screening (upon submission and then weekly) of all neonates in the NICU has been an implemented routine (with nasal and rectal swabs, as well as tracheal aspirates for intubated/tracheotomised neonates) since 2012.

The outbreak commenced in April 2020 and was resolved by June 2020. During that period, 10 neonates showed colonisation with ECC. Retained frozen samples of 10 of these strains were further analysed using NGS (see below). 

Preventive measures during the outbreak aimed to reduce nosocomial transmission and included contact precautions such as isolating MRGN neonates in single rooms whenever possible, using disposable gowns and nitrile gloves for the handling of all neonates and supervising the use of alcoholic hand disinfection (Desderman, Schülke & Mayr GmbH, Norderstedt, Germany). The dedicated assignment of specific nursing staff to the MRGN patient cohort was strictly implemented. Staff compliance with hygiene standards and environmental cleaning was also monitored. Despite conducting systematic line list analyses of medical and nursing staff and intensive microbiological examination of the extended patient environment, the results were inconclusive. However, a new standardised operating procedure was implemented to improve the handling of MRGN-colonised neonates by medical professionals and parents (see Supplementary Tables S1 and S2).

### 2.2. Strain Isolation and Microbiological Characterisation

All strains were isolated from the rectal swabs obtained for screening purposes as described previously [13]. Strain identification was done using MALDI–TOF MS (Bruker Daltonik GmbH, Bremen, Germany) and AST was performed using a VITEK 2 system (bioMérieux, Nürtingen, Germany). The latest EUCAST breakpoints (v10.0, 2020) were utilised for interpretation whenever possible.

### 2.3. Whole Genome Sequencing and Assembly

The genomic DNA for bacterial WGS was isolated from 2 mL of overnight culture in lysogeny broth (LB) using the GenElute Bacterial Genomic DNA kit (Sigma-Aldrich, Schnelldorf, Germany) according to the manufacturers’ instructions. The DNA was adjusted to 0.2 ng/µL in 10 mM of Tris-HCl pH 8.5 and 2.5 µL and subjected to tagmentation using the Nextera XT kit (Illumina, Berlin, Germany). The libraries were dual-indexed using PCR and the Nextera index set A (Illumina) and the mean fragment size was determined on a TapeStation 4200 instrument (Agilent, Waldbronn, Germany). The libraries were adjusted to 7 pM and subjected to 2 × 250 bp paired-end sequencing on a MiSeq instrument (Illumina, Berlin, Germany). Sequence data were demultiplexed according to the index sequences with the “Generate Fastq” workflow in MiSeq Reporter (Illumina) and are available in the National Centre for Biotechnology Information (NCBI, https://www.ncbi.nlm.nih.gov/ accessed on 12 October 2023) Sequence Read Archive (SRA) under BioProject PRJNA727521. The adapter sequences were removed, and the sequence data were filtered based on quality using BBDuk from BBMap v38.79 (Joint Genome Institute, https://sourceforge.net/projects/bbmap/ accessed on 12 October 2023). SKESA v2.3.0 [29] was applied for de novo assembly from filtered short reads. Species assignment was carried out based on the average nucleotide identity with GenBank type strains [30]. The assignment of isolates to *Enterobacter hormaechei* subspecies [31] was completed using MASH v2.2.2 [32] with a sketch size of 10,000 and k = 21 from the following reference genomes: CP017186.1 (subsp. *hoffmannii*), CP010377.1 (subsp. *hormaechei*), CP017180.1 (subsp. *oharae*), CP017179.1 (subsp. *steigerwaltii*) and CP017183.1 (subsp. *xiangfangensis*).

### 2.4. Phylogenetic Analysis

A contig-based whole-genome SNP alignment was done using PhaME v1.0.2 [33] and using ECC strain AR_0060 (GenBank: CP026719.1) as a reference. An approximately maximum likelihood (ML) tree was calculated for the isolates with FastTree 2 [34]. The tree was further processed and visualised using the GGTREE package for R [35].

### 2.5. Detection of Antimicrobial Resistance Genes and Mobile Genetic Elements

Genes conferring antimicrobial resistance (AMR) were identified in the assembled genomes using ABRicate v1.0.1 (Seemann T, https://github.com/tseemann/abricate accessed on 12 October 2023) and the NCBI AMRFinderPlus database (5684 entries after filtering, accessed 7 May 2021) [36]. The results were summarised with the summary option built into ABRicate and the isolates were clustered using an unweighted pair group method with arithmetic mean (UPGMA) based on a Jaccard distance matrix calculated with the antimicrobial-resistant gene presence using a custom script in R v4.2.2 [37]. MOB-suite [38] was used to detect mobile genetic elements (MGEs) as well as to reconstruct and type the plasmids from the de novo-assembled genomes [38,39,40].

## 3. Results

### 3.1. Patient Characteristics

We analysed perianal skin swabs from 6 male and 4 female neonates. The median gestational age at birth was 32 + 4 weeks (minimum 24 + 0, maximum 39 + 5 weeks). The interquartile range (IQR) of the birth weight was 787.5 to 2378 g with a median of 1585 g. The median chronological age at the onset of first MRGN detection was 21.5 days (IQR: 13 to 54.5 days). The median length of NICU stay was 60.5 days (IQR: 28.0 to 82.5 days).

Our retrospective analysis of NeoKiss bacterial surveillance revealed a significant increase in the total prevalence of 2MRGN-NeoPaed bacteria (20 cases per 100 neonates) in our NICU during the MRGN outbreak of April to June 2020. This was above the national median of 3.60 (75%-quantile 8.21) for that year. Additionally, the incidence of nosocomial-acquired multidrug-resistant bacteria (18.18 cases per 100 neonates) was also significantly higher than the national median of 3.12 cases (75%-quantile 7.52), with an incidence density of 6.93 nosocomial cases per 1000 patient days (national median: 1.02, 75%-quantile 2.19). In 2020, the majority of 2MRGN-NeoPaed (90.91%) and 3MRGN (100%) cases were acquired nosocomially (55 cases, 1443 patient days). Although the infection frequency for the identified MRGN colonisers was generally low in our NICU [13], two de novo nosocomial infections with 2MRGN-NeoPaed were identified in 2020, but they were not related to the analysed outbreak.

### 3.2. Characterization of Bacterial Isolates

Phenotypic AST and strain identification were carried out as part of the microbiological routine diagnostics. All isolates were identified as ECC since the current routine MALDI–TOF MS approaches fail to reliably identify these bacteria at the species level [41]. All isolates were classified as 2MRGN-NeoPaed based on phenotypic resistance to penicillins (piperacillin) and cephalosporins (ceftriaxon). Overall, the isolates exhibited a very homogeneous resistance pattern. This was also true for the minimal inhibitory concentrations (MIC) of fosfomycin (Fos) and tetracycline (Tet), where both antibiotics differed by only one dilution step, except for strains VA41244 and VA42547. These strains showed a lower MIC of ≤16 mg/L for Fos and isolate VA42547 was more susceptible to Tet having a MIC of ≤1 mg/L (Table 1).

**Figure 1:**
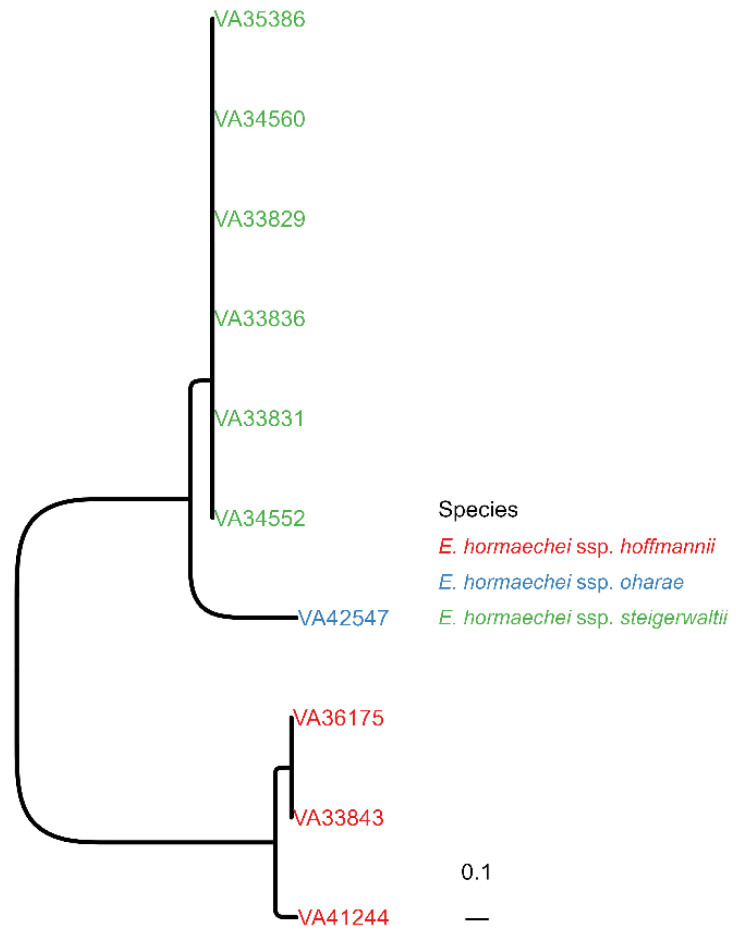
**Phylogenetic relationship between *E. hormaechei* isolates.** Approximately maximum likelihood (ML) tree from contig-based whole-genome single-nucleotide polymorphism (wgSNP) alignment is shown. The isolates were coloured according to their subspecies assignment.

For WGS, the genomes were constructed using de novo assembly from short reads resulting in a median of 39 contigs per isolate with a median of 111 for the mean read coverage (Table 2). The contigs were included in a wgSNP alignment with ECC strain AR_0060 as reference. The resulting approximately ML tree exhibited a topology with three clearly separated clades: The first clade consisted of six clonal isolates (VA33829, VA33831, VA33836, VA34552, VA34560, VA35386), the second of a single isolate (VA42547) and the third clade of two clonal strains (VA33843, VA36175) and one related isolate (VA41244) (Figure 1). In the next step, we determined the *Enterobacter* species and subspecies of all isolates based on the assembled genomes. Species identification was undertaken with MASH [32] calculating hashes based on the MinHash sketches derived from small oligonucleotides (*k*-mers), which can be very efficiently used for comparative analyses. Here, a set of *Enterobacter* species and subspecies reference genomes was used for comparison. We found all isolates were *Enterobacter hormaechei* including three subspecies: subsp. *steigerwaltii* (n = 6, 60%), subsp. *hoffmannii* (n = 3, 30%) and subsp. *oharae* (n = 1, 10%) (Table 2). As expected, subspecies assignment correlated perfectly with the three clades previously identified in the wgSNP-based approximately ML tree (Figure 1).

**Figure 2:**
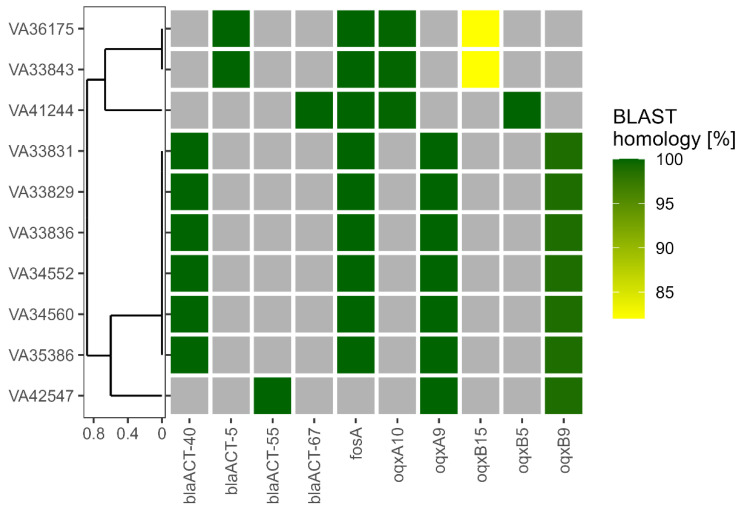
**Detection of antimicrobial resistance (AMR) genes.** AMR genes were identified in the assembled genomes of the isolates indicated in a Basic Local Alignment Search Tool (BLAST)-based approach. Coloured squares indicate the presence of the respective AMR gene with shading between yellow and green depending on BLAST homology. Gray squares show the absence of the respective AMR gene. The isolates were ordered according to a tree generated using unweighted pair group method with arithmetic mean (UPGMA) based on a Jaccard distance matrix derived from AMR gene presence/absence as shown here. Legend: blaACT gene—coding for a C-type beta-lactamase; fosA gene—coding for a glutathione-S-transferase that inactivates fosfomycin; oqxA gene—coding for a resistance-nodulation-cell division (RND) efflux pump conferring resistance to fluoroquinolone.

Further, the contigs of the bacterial isolates were screened for AMR genes, MGEs and plasmids. BLAST was used to screen for the presence of the AMR genes listed in the NCBI AMRFinderPlus database. Within the genomes of the *E. hormaechei* isolates, we identified a total of 10 AMR genes that had significant homologs (Figure 2). All isolates carried the class C β-lactamases responsible for conferring resistance against the penicillin/β-lactamase inhibitor combinations ampicillin/sulbactam and piperacillin/tazobactam, as well as against second- (Cefuroxime) and third (Ceftazidime)-generation cephalosporins (Table 1). Interestingly, the β-lactamase subtypes identified correlated with the subspecies. ACT-40 was found in all subsp. *steigerwaltii*, ACT-55 in the subsp. *oharae* while the two closely related subsp. *hoffmannii* (VA33843, VA36175) carried ACT-5 and the other subsp. *hoffmannii* (VA41244) ACT-67. Furthermore, genes for both subunits of the OqxAB multidrug resistance-nodulation-division (RND) efflux transporter were detected in all isolates. Here, for subsp. *oharae* and subsp. *Steigerwaltii*, the alleles *oqxA9*/*oqxB9* were detected, whereas for subsp. *hoffmannii* strains, different combinations of *oqxA10* with *oqxB15* (VA33843, VA36175) or *oqxB5* (VA41244) were found. Although two of the strains exhibited decreased resistance against fosfomycin (MIC ≤ 16 µg/mL, Table 1), the gene for Fos resistance-conferring glutathione transferase FosA was present in 9 out of 10 *E. hormaechei* isolates. Strain VA42547 (subsp. *oharae*) lacked the *fosA* gene, which was in accordance with its low fosfomycin MIC.

A significant concern lies in the dissemination of antibiotic resistances within hospital environments. Because mobile genetic elements (MGEs) such as plasmids, phages or transposons play a crucial role in the intercellular mobility of AMR genes [42], we identified MGEs and their spatial correlation with AMR genes in all isolates. An overview of the predicted plasmids and insertion (IS) elements is given in Supplementary Table S3. All isolates except VA34560 harboured a derivative of a small (~2.5 kb) plasmid of the ColE1 replicon type, which is widely distributed amongst *Enterobacter* and *Klebsiella*. Furthermore, we identified in all strains except VA41244 and VA42547 a larger (~4.7 kb) plasmid having the *mobBCD* mobilization genes only. Strain VA33843 carried in addition a large ~109 kb plasmid of the IncF replicon type, harbouring two IS elements with highest similarity to *E. hormaechei* subsp. *hormaechei* ECR091 plasmid pENT-4bd (accession: CP008907). A total of six IS elements were identified in strain VA41244: while one was encoded on plasmid p4, two were found on the genome, and four different IS elements on the larger plasmid p6. It is noteworthy that, based on the contig data, the identified AMR genes were neither found on the predicted plasmids nor in the vicinity of the chromosomal IS elements (Figure 2).

## 4. Discussion

The utilisation of whole-genome sequencing (WGS) and subsequent bioinformatic analysis has been widely accepted as an effective approach to strain typing in clinical microbiology and outbreak investigations [19,43,44], particularly for members of the Enterobacteriaceae family [19,45,46]. Therefore, we employed bacterial WGS in a neonatal intensive care unit (NICU) outbreak to establish the phylogenetic connection among the ECC isolates. This information served as a foundation for enhancing our isolation strategies moving forward.

While bacterial screening via environmental examinations (surface areas, sinks, etc.) was inconclusive, we were able to identify three *E. hormaechei* subspecies, mainly ssp. *steigerwaltii*, but also ssp. *hoffmannii* and a single case of ssp. *oharae*, as bacterial colonisers in our neonates. 

According to Sutton et al. [31], *E. hormaechei* is the most prevalent species among the clinical isolates of ECC. However, as mentioned earlier, distinguishing between different species within ECC using current routine MALDI–TOF MS systems is not feasible. This limitation could potentially be resolved by utilising enhanced databases [47].

Historically, *E. hormaechei* was suggested in 1989 as a new member of the *Enterobacter* genus [48]. During the last three decades, *E. hormaechei* has emerged as a relevant nosocomial pathogen in hospitalised adults [31], as well as an opportunistic pathogen in NICUs.

In 1997, Wenger et al. reported a case series involving six instances of *E. hormaechei* bloodstream infections and four cases of colonisation of preterm infants at the Hospital of the University of Pennsylvania, USA. The affected neonates had a low gestational age and birth weight. The transmission of *E. hormaechei* within the hospital setting may have been facilitated by environmental contamination, such as the contamination of neonatal incubator systems and doorknobs, as well as lapses in infection control practices among healthcare workers [49].

Da Silva et al. described six cases of neonates with *E. hormaechei* bloodstream infection in three NICUs in Rio de Janeiro, Brazil, in 2002 with complete recovery under antibiotic treatment. While the source remained unclear, parenteral nutrition was identified as the only common procedure [50]. In contrast, Dyabi et al. reported a series of three fatalities among five *E. hormaechei* bloodstream-infected neonates (mostly preterm) at the NICU of Mohamed VI University Hospital, Marrakesh, Morocco, in 2018. Most patients presented with respiratory distress as a common clinical sign [49].

Girlich et al. analysed a large incubator-related outbreak in the NICU of Bicêtre Hospital, Paris, France, with around 30% of neonates being physiologically colonised with ECC. Seven neonates had bacteraemia, and 6 of them were lost to fatal sepsis, systematically linked to (hypervirulent) *E. bugandensis* [51]. This study highlights the need for the better discrimination of *Enterobacter* species inside the ECC in this fragile population, as neonates colonised or infected with other ECC species had favourable outcomes in this study.

**Figure 3:**
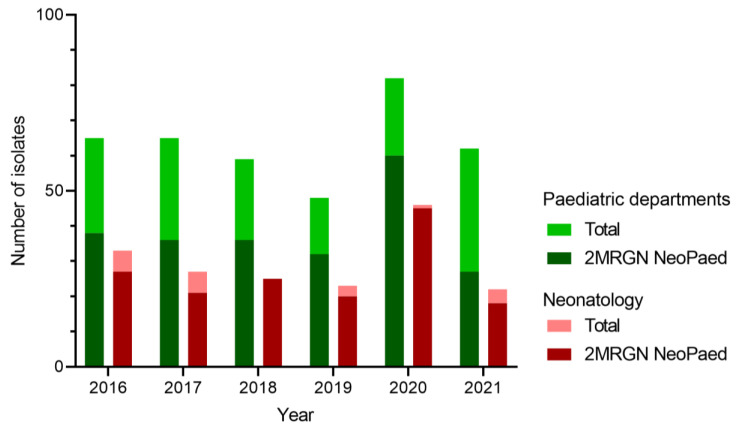
**Numbers of *E. cloacae* complex (ECC) isolates in screening materials per year.** In green, the number of annual ECC isolates from all paediatric departments of the University Hospital Erlangen are shown. In dark green the proportion of 2MRGN NeoPaed-classified ECC is depicted. Red shows similar data for the neonatology only.

In line with these reports, *E. hormaechei* colonisation in our study affected mostly preterm neonates during the neonatal period (<28 d). Although phylogenetic analyses revealed the involvement of four different clones, *E. hormaechei* subsp. *steigerwaltii* was the dominant outbreak clone (n = 6). However, the environmental contribution remained inconclusive and general improvement of infection control techniques among health workers and parents (see Supplementary Files) resolved the issue. While the outbreak-driven increase in ECC screening isolates was evident in 2020, we observed a drop to normal levels in the following year (Figure 3). Therefore, this aspect seems to be an important mainstay of hospital hygiene regarding the sustainable containment of ECC in general and particularly of *E. hormaechei*. It has been shown that microbial communities on surfaces in patient rooms closely resemble the respective skin microbiome [26,52]. In NICUs, the predominant bacterial genera were *Streptococcus* and *Staphylococcus* [53]. Martineau et al. also pointed out the strong effect of surface disinfection on bacterial communities in the case of *Serratia marcescens* [19], as also described by others [28]. The fact that we did not identify these bacterial communities in our patient environment might imply sufficient surface hygiene, further supporting the human-to-human transmission of *E. hormaechei* during our outbreak despite inconclusive line list analyses.

Outbreak investigations of nosocomial infections help to limit the healthcare burden of this increasing threat. In this study, we aimed to extend the knowledge on *E. hormaechei* gathered by the above-mentioned patient reports via implanting bacterial WGS. Our results provided extended information on the phylogenetic relationship and presence of AMR genes, MGEs and plasmids. The detection of class C β-lactamase genes in all isolates correlated with the MDR phenotype, which led to their classification as 2MRGN NeoPaed. However, despite 9 of 10 strains carrying the *fosA* gene, two isolates exhibited a reduced fosfomycin MIC. The reason for the lower Fos MIC in isolate VA41244 could be lack of expression of a functional FosA protein under the conditions used for AST [54]. The identification of AMR genes in bacterial WGS data is a very valuable approach to investigating MDR strains and the possible transfer of these genes to other bacteria in a clinical setting [55]. The transfer of AMR genes within a species but also across species boundaries can occur via horizontal gene transfer (HGT). The main routes of HGT in bacteria are conjugation, transduction, and the uptake of free DNA (natural transformation). The transfer of AMR genes via HGT is much more efficient if these genes are linked to MGEs, e.g., plasmids, phages or IS elements [56]. To investigate the potential of our strains to transfer AMR through HGT, we reconstructed their plasmids from short-read data. With this approach, we were able to assemble two plasmids of ~2.5 kb and ~4.7 kb length to circular contigs for the majority of the isolates. The reconstruction of the other plasmids was less efficient and would have required long-read sequencing techniques for improvement [57]. Nonetheless, we were able to assign the contigs to their plasmid origin with none of the AMR genes present on these fragments. Furthermore, because none of the AMR genes were linked to a contig harbouring an MGE, we considered all our *E. hormaechei* isolates to have a low potential to transfer these genes to other bacteria in a clinical setting.

## 5. Conclusions and Future Aspects

Our findings highlight the importance of implementing WGS as an effective approach to preventing and controlling bacterial infections in NICUs. The use of WGS has the capacity to revolutionise the way outbreak investigations are conducted in healthcare settings. It will improve the knowledge of bacterial ecology and uncover nosocomial transmission pathways and chance clusters. Furthermore, WGS provides pertinent clinical information regarding antimicrobial resistance and virulence genes that may not be detected by other testing methods. 

Although WGS may eventually replace other molecular genomic methods as the main tool for molecular subtyping, it will continue to serve as a supplementary tool for traditional surveillance and epidemiological investigations, such as evaluating transmission points. While the robustness and scalability of WGS to accurately identify bacterial strains significantly improves patient care, several obstacles need to be addressed to routinely implement whole-genome sequencing (WGS) for outbreak investigations. These barriers include the turnaround time, accessibility, cost, and standardisation, as discussed by Sansom et al. [58]. In the future, WGS might emerge as the primary genomic surveillance tool with prospective potential [59], enabling the early identification of potential transmissions. This could contribute to a reduction in the morbidity and mortality rates associated with outbreaks.

## Figures and Tables

**Table 1:** Minimal inhibitory concentration (MIC) in mg/L for fosfomycin (Fos) and tetracycline (Tet) of the isolates.

Isolate	Fos	Tet
VA33829	32	4
VA33831	32	2
VA33836	64	2
VA33843	64	4
VA34552	32	2
VA34560	32	4
VA35386	32	2
VA36175	64	4
VA41244	≤16	2
VA42547	≤16	≤1

**Table 2:** Assembly results, subspecies assignment and genome accessions of *E. hormaechei* isolates.

Isolate	Number of Contigs	Mean Read Coverage	*E. hormaechei* Subspecies	Sketches Identified (MASH)	Genome Accession
VA33829	22	111	*steigerwaltii*	8245	JAHBEC000000000
VA33831	33	64	*steigerwaltii*	8243	JAHBEB000000000
VA33836	22	138	*steigerwaltii*	8245	JAHBEA000000000
VA33843	71	106	*hoffmannii*	8293	JAHBDZ000000000
VA34552	36	125	*steigerwaltii*	8245	JAHBDY000000000
VA34560	24	109	*steigerwaltii*	8243	JAHBDX000000000
VA35386	42	98	*steigerwaltii*	8245	JAHBDW000000000
VA36175	77	127	*hoffmannii*	8294	JAHBDV000000000
VA41244	167	111	*hoffmannii*	8292	JAHBDU000000000
VA42547	112	118	*oharae*	7148	JAHBDT000000000

## Data Availability

All bacterial datasets can be accessed via BioProject ID PRJNA727521 through the NCBI SR-archive. Additional information can be obtained from the corresponding author upon reasonable request.

## Supplementary Materials

The following supporting information can be downloaded at: https://www.mdpi.com/article/10.3390/children10101696/s1; Tables S1 and S2: Standardised operating procedure for improved handling of MRGN-colonised neonates by medical professionals and parents: Table S1: NICU hygiene measures regarding handling of neonates and mothers colonized with multiresistant bacteria (MRB) and Serratia marcescens (SERMA). Table S2: Breastfeeding and Kangaroo care in neonates with positive bacterial screening for multiresistant bacteria (MRB) and Serratia marcescens (SERMA). Table S3: Overview of the predicted plasmids and insertion elements.
