# Supplementary material for: Application of Next-Generation Sequencing to Enterobacter Hormaechei Subspecies Analysis during a Neonatal Intensive Care Unit Outbreak
Source: Children (Basel). 2023 Oct 16;10(10):1696. doi: 10.3390/children10101696 (PMC10605273; doi:10.3390/children10101696)
Supplement: Supplementary file 1 [file children-10-01696-s001.zip › children-2645518-supplementary.pdf]

Supplementary Material

Application of Next-Generation Sequencing to Enterobacter Hormaechei Subspecies Analysis during a Neonatal Intensive Care Unit Outbreak

Patrick Morhart [1,†], Roman G. Gerlach [2,†], Caroline Kunz [2], Jürgen Held [2], Giuseppe Valenza [2], Joachim Wölfle [3], Heiko Reutter [1], Gregor J. Hanslik [1] and Fabian B. Fahlbusch [4,*]

[1] Division of Neonatology and Paediatric Intensive Care Medicine, Department of Paediatrics and Adolescent Medicine; Friedrich-Alexander-University of Erlangen-Nürnberg, 91054 Erlangen, Germany; patrick.morhart@uk-erlangen.de (P.M.); heiko.reutter@uk-erlangen.de (H.R.); gregor.hanslik@uk-erlangen.de (G.J.H.)

[2] Institute of Microbiology—Clinical Microbiology, Immunology and Hygiene; Friedrich-Alexander-University of Erlangen-Nürnberg, 91054 Erlangen, Germany; caroline.kunz@gmx.net (C.K.); juergen.held@uk-erlangen.de (J.H.); giuseppe.valenza@uk-erlangen.de (G.V.)

[3] Department of Paediatrics and Adolescent Medicine, Friedrich-Alexander-University of Erlangen-Nürnberg, 91054 Erlangen, Germany; joachim.woelfle@uk-erlangen.de

[4] Neonatology and Pediatric Intensive Care, Faculty of Medicine, University of Augsburg, 86156 Augsburg, Germany

* Correspondence: fabian.fahlbusch@uk-augsburg.de

† These authors contributed equally to this work.

Table S1	NEONATES			MOTHERS
	**2MRGN-NeoPaed**	**3 or 4MRGN, MRSA, VRE**	**SERMA**	**2, 3 or 4MRGN, VRE, MRSA with negative neonate.**
**Patient room**	Parents must wear DG.	**24 h-RI**: Parents do not need to wear DG; consequent HD required. **No 24 h-RI**: Parents must wear DG.	Parents must wear DG. No 24 h-RI allowed.	Parents must wear disposable gowns. No 24 h-RI allowed.
**Breastfeeding**	yes	yes (certain limitations apply - see Table S2, 4MRGN)	yes (certain limitations apply – see Table S2, 4MRGN)	With 2MRGN-NeoPaed + VRE: yes. With 3MRGN + 4MRGN, MRSA: no.
**Kangaroo care**	yes	yes (certain limitations apply - see Table S2, 4MRGN)	yes - however not in case of a breakout (see suppl. Table S2, 4MRGN)	With VRE, 2MRGN-NeoPaed + 3MRGN: yes. With 4MRGN + MRSA: no.
**When parents leave the patient room**	DG, Dispose gown, HD.	**24 h-Rooming-in:** Parents must put on disposable gowns. **No 24h-Rooming in:** Dispose gown, hand disinfection.	Dispose Gown, HD. During a breakout: Replace disposable gown by new one.	Dispose Gown, HD. Minimize potential contact points outside the patient room. 3MRGN +4MRGN+MRSA: Replace DG by new one.

Table S1. NICU hygiene measures regarding handling of neonates and mothers colonized with multiresistant bacteria (MRB) and Serratia marcescens (SERMA). Breastfeeding of mothers with evidence of MRB/child (still) negative: Since a routine examination of every portion of breast milk was not feasible with reasonable effort in everyday clinical practice, breast milk was preliminary discarded in this scenario. An exemption was made for 2MRGN and VRE. If necessary, breastfeeding was stopped depending on the mother's wishes. Definition of an outbreak scenario: two or more nosocomial infections in which an epidemiologic connection is likely or suspected (§6 Abs. 3, German Infection Protection Act, IfSG). Legend: DG= disposable gown (i.e., longsleeve gown covering upper and lower body), RI= rooming-in, HD= hand disinfection, Legend: MRGN= multidrug-resistant gram-negative bacteria, MRSA= Methicillin-resistent staphylococcus aureus, VRE= Vancomycin-resistent Enterococcus.

Table S2	Neonate with positive bacterial screening			
	**Multiresistant bacteria**			**SERMA**
	**2MRGN-NeoPaed**	**3MRGN, MRSA, VRE**	**4MRGN**	
**Breastfeeding**	yes	yes	yes, but only after prior education about potiential infection risk and sufficient maternal compliance, e.g. hand disinfection	yes - regulation for 4MRGN applies
**Kangaroo care**	yes	yes	yes, but only after prior education about potiential infection risk and sufficient maternal compliance, e.g., hand disinfection	individual detection: yes<br>outbreak scenario: no

Table S2. Breastfeeding and Kangaroo care in neonates with positive bacterial screening for multiresistant bacteria (MRB) and Serratia marcescens (SERMA). Legend: MRGN= multidrug-resistant gram-negative bacteria, MRSA= Methicillin-resistent staphylococcus aureus, VRE= Vancomycin-resistent Enterococcus. Disposable gown= longsleeve gown covering upper and lower body.

Isolate	Genetic element	Plamid size	Plasmid contig number(s)	MGE (contig number)	AMR genes (contig number)
VA33829	genome				oqxA9B9 (18), fosA (22), blaCT-40 (22)
	p1	2495 bp	1 (circular)		
	p2	4665 bp	2 (circular)		
VA33831	genome				oqxA9B9 (2), fosA (33), blaCT-40 (33)
	p1	2495 bp	1 (circular)		
	p2	3713 + 709 bp	6, 27		
VA33836	genome				oqxA9B9 (2), fosA (13), blaCT-40 (13)
	p1	2346 bp	6		
	p2	4665 bp	1 (circular)		
VA33843	genome			ISKpn34 (43)	oqxA10B15 (11), fosA (29), blaCT-5 (16)
	p1	2495 bp	1 (circular)		
	p2	4665 bp	2 (circular)		
	p3	108558 bp	42, 45, 47, 66	ISBrsa1 (45), ISKpn38 (66)	
VA34552	genome				oqxA9B9 (13), fosA (16), blaCT-40 (18)
	p1	2463 bp	21		
	p2	3635 bp	36		
VA34560	genome				oqxA9B9 (12), fosA (24), blaCT-40 (17)
	p1	3381 bp	20		
VA35386	genome				oqxA9B9 (12), fosA (41), blaCT-40 (19)
	p1	595 + 710 + 812 + 1073 bp	17, 16, 4, 21		
VA36175	genome				oqxA10B15 (35), fosA (29), blaCT-5 (17)
	p1	2495 bp	1 (circular)		
	p2	4665 bp	2 (circular)		
VA41244	genome			IS904 (92), IS5708 (97)	oqxA10B5 (52), fosA (60), blaCT-67 (100)
	p1	2494 bp	1 (circular)		
	p2	4307 bp	2 (circular)		
	p3	8814 bp	79, 152		
	p4	14103 bp	46, 157	IS903 (46)	
	p5	21597 bp	86, 99		
	p6	49039 bp	66, 72, 91, 107, 135, 146, 153	ISEcl1 (66), ISKpn26 (91), ISKpn34 (135), ISEcl1 (135)	
VA42547	genome			IS903B (78)	oqxA9B9 (8), blaCT-55 (111)
	p1	2495 bp	1 (circular)		
	p2	5405 bp	2, 56	ISKpn26 (56)	

Table S3. Overview of predicted plasmids. Legend: Plasmid size= either predicted total plasmid size or sum of individual contig sizes likely resembling one plasmid, Contigs= contigs forming the plasmid and whether the assembly is circular. Legend: MGE= mobile genetic elements, AMR= antimicrobial resistance, blaACT gene – coding for a C-type beta-lactamase; fosA gene – coding for a glutathione-S-transferase that inactivates fosfomycin; oqxA gene – coding for a resistance-nodulation-cell division (RND) efflux pump conferring resistance to fluoroquinolone.
